# Hospital ethical climate and nurses’ cyber ethics literacy: the mediating role of compassion competence

**DOI:** 10.3389/fmed.2026.1876044

**Published:** 2026-07-31

**Authors:** Yanmin Xue, Ruixin Ding, Yi Lu, Xiyu Wang, Zhongning Zhou, Mingxia Li

**Affiliations:** 1 School of Nursing, Shandong University of Traditional Chinese Medicine, Jinan, China; 2 Otorhinolaryngology Department, The 80th Group Army Hospital of the People’s Liberation Army, Weifang, China

**Keywords:** compassion competence, cyber ethics literacy, hospital ethical climate, nurses, social cognitive theory

## Abstract

**Objective:**

This study examined the current level of nurses’ cyber ethics literacy in tertiary hospitals in China and explored its associations with perceived hospital ethical climate and nurses’ compassion competence. A theory-driven, non-causal mediation model was used to examine whether compassion competence statistically mediated the association between hospital ethical climate and cyber ethics literacy.

**Methods:**

A multicentre cross-sectional survey was conducted between September and November 2024. Guided by social cognitive theory, nurses’ cyber ethics literacy, hospital ethical climate, and compassion competence were measured using the Nurses’ Cyber Ethics Literacy Questionnaire, the Hospital Ethical Climate Scale, and the Compassion Competence Scale for Nurses, respectively, to analyze the correlations among the three variables.

**Results:**

Nurses’ cyber ethics literacy was at a moderately high level (136.82 ± 23.89). Cyber ethics literacy was positively correlated with perceived hospital ethical climate (*r* = 0.370, *p* < 0.01) and compassion competence (*r* = 0.290, *p* < 0.01). In the theoretical structural equation model, compassion competence partially mediated the association between hospital ethical climate and cyber ethics literacy, with the standardized indirect effect accounting for 26.08% of the total effect.

**Conclusion:**

Hospital ethical climate and compassion competence were positively associated with nurses’ cyber ethics literacy. The mediation result is compatible with the proposed Social Cognitive Theory framework, suggesting that a supportive ethical environment may be linked to better cyber ethics literacy partly through stronger compassion competence. Because the study was cross-sectional and based on self-report data, causal inferences cannot be drawn.

## 1 Introduction

With the deep integration of Internet Plus Healthcare, digital technology has become an important tool for improving the efficiency, accessibility, and continuity of healthcare services (1). The widespread use of social media, telehealth, remote monitoring, and online patient communication has strengthened information exchange and clinical collaboration (2, 3). At the same time, these technologies have blurred the boundaries between personal and professional life and have created ethical challenges such as privacy breaches, inappropriate disclosure, online incivility, misinformation, and unclear professional boundaries (4, 5). The World Health Organization’s global strategy on digital health emphasizes that digital health governance should address safety, security, privacy, interoperability, and ethical use of data (6). In this context, nurses need not only technical proficiency but also sound ethical judgment in digital environments. Therefore, nurses’ cyber ethics literacy has become an important component of digital nursing professionalism.

Cyber ethics literacy refers to the moral values, norms, knowledge, and behavioral standards needed to act responsibly in online environments, including privacy protection, respect for intellectual property, responsible information sharing, and protection of the accuracy and reliability of health-related information (7). In international health-professional literature, the related concept of e-professionalism is often used to describe the attitudes, values, and behaviors that healthcare professionals demonstrate online to maintain professional integrity and public trust (8). Ethical misconduct by healthcare professionals in online settings may contribute to privacy breaches, misuse of patient information, cyber incivility, and reputational or legal consequences (9–13). Therefore, nurses need to extend traditional ethical principles, such as respect, confidentiality, beneficence, and accountability, to digital clinical and social environments.

Compassion competence and empathy are related but distinct constructs. Empathy mainly refers to understanding or sharing another person’s emotional state. Compassion competence is broader: it includes empathic understanding as well as sensitivity to suffering, communication competence, insight, motivation, and the capacity to respond appropriately to patients’ needs (14). In the present study, the central mediator is nurses’ compassion competence, as measured by the Compassion Competence Scale for Nurses. Although some Chinese literature has used the term empathy when discussing this scale, the manuscript uses compassion competence consistently because the instrument was designed to capture a broader action-oriented professional capacity rather than empathy alone.

In nursing practice, compassion competence may support ethical judgment by helping nurses identify patient vulnerability, anticipate possible harm, and respond to distress in a respectful and patient-centered manner (15, 16). In digital healthcare environments, nurses with higher compassion competence may be more sensitive to patients’ privacy expectations, emotional reactions to online communication, and potential consequences of public or semi-public digital behaviors (17). This provides a conceptual basis for expecting compassion competence to be associated with cyber ethics literacy.

Hospital ethical climate refers to nurses’ shared perceptions of what constitutes ethically appropriate conduct and how ethical issues are handled in their work setting (18, 19). A favorable ethical climate may provide moral cues, role expectations, and organizational support that encourage ethical reflection and professional behavior. Previous studies have shown that the ethical climate is associated with nurses’ ethical conduct and service behavior (20, 21). Ethical climate may also shape nurses’ compassion competence by reducing moral distress, supporting respectful communication, and providing emotional and organizational resources for compassionate practice (22). However, in the online environment, the interaction between nurses’ compassion competence and the hospital’s ethical atmosphere on nurses’ cyber ethics literacy has not been fully explored through empirical research.

Social cognitive theory provides a suitable framework for the present study because it emphasizes reciprocal relationships among environmental factors, personal factors, and behavior (23, 24). From this perspective, perceived hospital ethical climate can be conceptualized as an environmental factor, compassion competence as a personal professional capacity, and cyber ethics literacy as a behavioral-cognitive outcome in digital healthcare. We specified a mediation model rather than a moderation model because the theory suggests that the organizational environment may shape personal capacities that are subsequently associated with professional behavior. Nevertheless, given the cross-sectional design, the mediation model is theoretical and exploratory rather than causal. This study therefore aimed to examine: (1) the current level of nurses’ cyber ethics literacy; (2) the associations among hospital ethical climate, compassion competence, and cyber ethics literacy; and (3) whether compassion competence statistically mediates the association between hospital ethical climate and cyber ethics literacy in a theory-driven structural equation model.

## 2 Methods

### 2.1 Study design

This study used a multicenter cross-sectional quantitative survey design. The reporting and interpretation of mediation analyses were framed as theory-driven and non-causal because all data were collected at a single time point.

### 2.2 Study setting and sampling

Participants were recruited from three Grade A tertiary hospitals in Shandong Province, China, between September and November 2024 using convenience sampling. The study was described as multicenter because clinical nurses were recruited through the nursing departments of three hospitals. To protect participant anonymity, hospital identifiers were not retained in the analytic dataset; therefore, hospital-specific participant counts and hospital-level comparisons could not be reported. This issue is acknowledged as a limitation.

Inclusion criteria were: (1) possession of a valid nursing license; (2) current engagement in clinical nursing practice; (3) normal communication, comprehension, and expression abilities; and (4) voluntary participation. Exclusion criteria was: Clinical nurses on leave or attending off-site training.

According to Kendall’s sample size calculation method, the sample size should be 5–10 times the number of variables (25). Structural equation modeling generally requires an adequate sample size, and a sample of approximately 200 or more is commonly considered acceptable for many applied SEM models (26). The study included 22 independent variables. After allowing for an estimated 20% invalid or non-response rate and considering the requirements for structural equation modeling, the minimum target sample size was 264. A total of 479 questionnaires were distributed. After removing 72 invalid questionnaires due to straight-line or obviously patterned responses, 407 valid questionnaires were retained for the final analysis. The valid response rate was 85.0% (calculated as 407/479 × 100%).

### 2.3 Measures

#### 2.3.1 Demographic information questionnaire

The demographic questionnaire was developed by the research team and included gender, age, marital status, educational attainment, years of service, professional title, employment relationship, weekly working hours, monthly personal income, prior training in cyber ethics, and average daily internet use.

#### 2.3.2 Nurses’ cyber ethics literacy questionnaire

The Nurses’ Cyber Ethics Literacy Questionnaire was developed by Ding et al. based on an evaluation index system for clinical nurses’ cyber ethics literacy (27). It contains 34 items across three dimensions: knowledge of cyber ethics and law, beliefs in cyber ethics, and cyber ethical behavior. Items are scored on a 5-point Likert scale from 1 (completely disagree) to 5 (completely agree), with total scores ranging from 34 to 170. Higher scores indicate higher cyber ethics literacy. The development study reported a Cronbach’s alpha of 0.976 for the overall questionnaire and 0.925, 0.956, and 0.975 for the three dimensions, respectively; split-half reliability was 0.880. Content validity was satisfactory, with I-CVI values ranging from 0.875 to 1.000 and S-CVI = 0.912. Exploratory factor analysis showed KMO = 0.966 and Bartlett’s test *p* < 0.001; the extracted factors explained 73.116% of the total variance. In the present sample, Cronbach’s alpha was 0.970. Because this instrument remains newly developed and independent external validation is still limited, additional psychometric evidence was reported from the present measurement model and this limitation was acknowledged.

#### 2.3.3 Compassion competence scale for nurses

The Compassion Competence Scale for Nurses is a 17-item instrument originally developed by Lee and Seomun and later translated for use among Chinese nurses (14, 28). It includes three dimensions: communication competence (8 items), sensitivity (5 items), and insight competence (4 items). Items are scored on a 5-point Likert scale from 1 (strongly disagree) to 5 (strongly agree). Total scores range from 17 to 85, with higher scores indicating stronger compassion competence. The Chinese version showed a Cronbach’s alpha of 0.870, with subscale coefficients ranging from 0.700 to 0.790 (28). In the present study, Cronbach’s alpha was 0.954.

#### 2.3.4 Hospital ethical climate scale

The Hospital Ethical Climate Scale is a 25-item instrument for measuring nurses’ perceptions of hospital ethical climate, including its Chinese adaptation (19, 29). It contains five dimensions: nurses, patients, physicians, administrators, and hospital. Items are scored on a 5-point Likert scale from 1 (not at all) to 5 (completely), with total scores ranging from 25 to 125. Higher scores indicate a more favorable perceived hospital ethical climate. The Chinese version reported a Cronbach’s alpha of 0.915 (29). In this study, Cronbach’s alpha was 0.948.

### 2.4 Data collection

Data were collected using both electronic and paper questionnaires. Electronic questionnaires were administered through Questionnaire Star,^1^ a widely used survey platform in mainland China. With the consent and assistance of hospital nursing departments, researchers distributed questionnaires to eligible clinical nurses. For electronic responses, each IP address and device were limited to one submission, and all questions were mandatory before submission. After data collection, two graduate students checked questionnaire completeness and response quality. We excluded invalid questionnaires containing straight-line responses, internally inconsistent answers, or obviously patterned response sequences. Out of the 479 distributed questionnaires that underwent screening, 72 were excluded, and 407 valid questionnaires were retained for the final analysis, resulting in a valid response rate of 85.0%.

### 2.5 Data analysis

Data were analyzed using SPSS 26.0 and AMOS 28.0. Descriptive statistics were used to summarize participants’ characteristics and scale scores. Continuous variables are presented as mean ± standard deviation, and categorical variables are presented as frequency and percentage. Data were considered approximately normally distributed when skewness coefficients were within ±3 and kurtosis coefficients were within ±10.35; the main scale scores met these criteria (30).

Pearson correlation analysis was used to examine bivariate associations among hospital ethical climate, compassion competence, and cyber ethics literacy. Correlations between the total cyber ethics literacy score and its subdimension scores were reported descriptively only and were not interpreted as independent evidence because the subdimensions are components of the total score.

Multiple linear regression was used to explore factors associated with nurses’ cyber ethics literacy. Categorical variables with more than two categories were dummy-coded. The reference categories were: education level = associate degree or below; professional title = nurse. Prior training in cyber ethics was coded as 0 = no and 1 = yes. Years of service, weekly working hours, monthly personal income, and average daily internet use were entered as ordinal category scores according to the categories shown in Table 1 and were interpreted as trend variables. Hospital ethical climate and compassion competence were entered as continuous total scores. Multicollinearity was assessed using tolerance and variance inflation factor values.

**Table 1:** Demographic characteristics of participants (*n* = 407).

Variable	Category	*n*	%
Gender	Female	364	89.4
	Male	43	10.6
Age (years)	18–25	90	22.1
	26–30	141	34.6
	31–40	140	34.4
	41–50	34	8.4
	≥51	2	0.5
Marital status	Unmarried	153	37.6
	Married	250	61.4
	Divorced/widowed	4	1.0
Education level	≤Associate degree	55	13.5
	Bachelor’s degree	337	82.8
	Master’s degree or above	15	3.7
Years of service	≤3	114	28.0
	4–10	151	37.1
	11–15	115	28.3
	16–20	21	5.1
	≥21	6	1.5
Professional title	Nurse	120	29.5
	Senior nurse	170	41.8
	Supervisor nurse	99	24.3
	Associate chief nurse or above	18	4.4
Employment relationship	Contract-based	216	53.1
	Personnel agency	34	8.3
	Labor dispatch	103	25.3
	Regular staff	54	13.3
Weekly working hours	<40	30	7.4
	40–45	209	51.3
	46–50	85	20.9
	>50	83	20.4
Monthly personal income	<3,000	28	6.9
	3,000–6,000	89	21.8
	6,001–9,000	236	58.0
	9,001–12,000	45	11.1
	>12,000	9	2.2
Training in cyber ethics	Yes	158	38.8
	No	249	61.2
Average daily internet use (hours)	<1	76	18.7
	1–3	239	58.7
	3–5	63	15.5
	>5	29	7.1

A structural equation model was constructed based on social cognitive theory, with hospital ethical climate as the environmental factor, compassion competence as the personal factor, and cyber ethics literacy as the outcome. The five dimensions of the Hospital Ethical Climate Scale, the three dimensions of the Compassion Competence Scale for Nurses, and the three dimensions of the Nurses’ Cyber Ethics Literacy Questionnaire were modeled as observed indicators of their respective latent variables. Maximum likelihood estimation was used because the dimension-level scores were approximately normally distributed and treated as continuous indicators (26). Bootstrap resampling with 5,000 samples was used to estimate the indirect effect and 95% confidence interval was reported, and the proportion mediated was reported as a descriptive mediation effect-size index (25). Model fit was evaluated using *χ*^2^/df, GFI, AGFI, NFI, IFI, TLI, CFI, and RMSEA. Because hospital identifiers were not retained in the anonymized analytic dataset, cluster-robust or multilevel models could not be performed; this limitation was explicitly considered when interpreting statistical significance.

To reduce common method bias, participation was anonymous and voluntary, respondents were informed that there were no right or wrong answers, and the questionnaire was used only for research purposes. However, because all main variables were self-reported and collected at the same time, common method variance could not be ruled out. This issue was added to the limitations in accordance with recommendations on common method bias in behavioral research (31).

### 2.6 Ethical considerations

This study was reviewed and approved by the Affiliated Hospital of Shandong University of Traditional Chinese Medicine (No. 2022-0018). All participants were informed of the study’s purpose, the voluntary nature of participation, anonymity, and the right to withdraw at any time. Written informed consent was obtained for paper questionnaires, and electronic informed consent was obtained before online questionnaire completion. The collected data were used solely for this study.

## 3 Results

### 3.1 Participant characteristics and questionnaire flow

**Figure 1:**
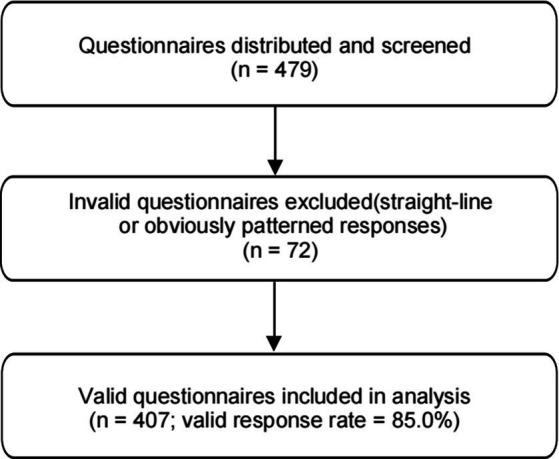
Participant flow diagram.

A total of 479 questionnaires were distributed and screened. 72 were excluded due to straight-line or obviously patterned responses, and 407 valid questionnaires were included in the final analysis (valid response rate = 85.0%; Figure 1). Among the 407 participants, 364 were women (89.4%) and 43 were men (10.6%). Most participants held a bachelor’s degree (82.8%), 41.8% held the professional title of senior nurse, and 61.2% had not received training in cyber ethics. Detailed characteristics are shown in Table 1.

### 3.2 Scores of nurses’ cyber ethics literacy, hospital ethical climate, and compassion competence

The total mean score for nurses’ cyber ethics literacy was 136.82 ± 23.89, indicating a moderately high level. The mean item score was highest for cyber ethical behavior (4.06 ± 0.83) and lowest for knowledge of cyber ethics and law and beliefs in cyber ethics (both 3.99 ± 0.86). The mean total scores for hospital ethical climate and compassion competence were 109.98 ± 12.04 and 71.77 ± 13.29, respectively (Table 2).

**Table 2:** Scores of nurses’ cyber ethics literacy, hospital ethical climate, and compassion competence.

Variable	Score range	Number of items	Total score (Mean ± SD)	Mean item score (Mean ± SD)
Knowledge of cyber ethics and law	6–30	6	23.93 ± 5.16	3.99 ± 0.86
Beliefs in cyber ethics	11–55	11	43.88 ± 9.48	3.99 ± 0.86
Cyber ethical behavior	17–85	17	69.01 ± 14.13	4.06 ± 0.83
Nurses’ cyber ethics literacy	34–170	34	136.82 ± 23.89	4.02 ± 0.70
Nurse	4–20	4	17.80 ± 2.35	4.45 ± 0.59
Patient	4–20	4	17.67 ± 2.31	4.42 ± 0.58
Physician	5–25	5	21.51 ± 3.29	4.30 ± 0.66
Administrator	6–30	6	26.63 ± 3.82	4.44 ± 0.64
Hospital	6–30	6	26.37 ± 3.58	4.39 ± 0.60
Hospital ethical climate	25–125	25	109.98 ± 12.04	4.40 ± 0.48
Communication competence	8–40	8	34.03 ± 7.26	4.25 ± 0.91
Sensitivity	5–25	5	21.11 ± 4.88	4.22 ± 0.98
Insight competence	4–20	4	16.63 ± 4.00	4.16 ± 1.00
Nurses’ compassion competence	17–85	17	71.77 ± 13.29	4.22 ± 0.78

The mean item score was calculated by dividing the total score by the number of items; the corresponding SD was recalculated using the same denominator.

### 3.3 Correlations among nurses’ cyber ethics literacy, hospital ethical climate, and compassion competence

Pearson correlation analysis showed that nurses’ cyber ethics literacy was positively correlated with hospital ethical climate (*r* = 0.370, *p* < 0.01) and compassion competence (*r* = 0.290, *p* < 0.01). Hospital ethical climate was also positively correlated with compassion competence (*r* = 0.399, *p* < 0.01). Correlations between cyber ethics literacy and its own subdimensions are presented for completeness but were not used as independent evidence because the subdimensions constitute the total score (Table 3).

**Table 3:** Correlations among nurses’ cyber ethics literacy, hospital ethical climate, and compassion competence.

Variable	1	2	3	4	5	6
1. Nurses’ cyber ethics literacy	1					
2. Knowledge of cyber ethics and law	0.642^**^	1				
3. Beliefs in cyber ethics	0.814^**^	0.404^**^	1			
4. Cyber ethical behavior	0.910^**^	0.449^**^	0.558^**^	1		
5. Hospital ethical climate	0.370^**^	0.422^**^	0.289^**^	0.277^**^	1	
6. Nurses’ compassion competence	0.290^**^	0.352^**^	0.206^**^	0.224^**^	0.399^**^	1

^**^p < 0.01.

### 3.4 Factors associated with nurses’ cyber ethics literacy

Multiple linear regression was performed with nurses’ cyber ethics literacy as the dependent variable. Independent variables included demographic and work-related variables that showed significant differences in preliminary analyses, as well as hospital ethical climate and compassion competence. The model explained a modest proportion of the variance in cyber ethics literacy (*R*^2^ = 0.206, adjusted *R*^2^ = 0.182, *p* < 0.001). After adjustment, hospital ethical climate (*β* = 0.273, *p* < 0.001), compassion competence (*β* = 0.129, *p* = 0.010), and master’s degree or above (*β* = 0.122, *p* = 0.031) were statistically associated with cyber ethics literacy. Prior training in cyber ethics was not statistically significant in this model (*p* = 0.232); therefore, training is discussed as a practical and theoretical implication rather than as a significant predictor in the present regression model. All VIF values were below 10, indicating no serious multicollinearity (Table 4).

**Table 4:** Multiple linear regression analysis of factors associated with nurses’ cyber ethics literacy.

Variable	B	SE	*β*	*t*	*p*	Tolerance	VIF
Constant	57.052	12.279		4.646	<0.001		
Bachelor’s degree	3.948	3.180	0.062	1.242	0.215	0.797	1.254
Master’s degree or above	15.423	7.110	0.122	2.169	0.031	0.640	1.563
Years of service	−0.363	1.344	−0.014	−0.270	0.787	0.722	1.384
Senior nurse	−2.922	2.769	−0.060	−1.055	0.292	0.615	1.625
Supervisor nurse	3.754	3.315	0.067	1.132	0.258	0.567	1.763
Associate chief nurse or above	3.140	6.557	0.027	0.479	0.632	0.632	1.583
Weekly working hours	−0.962	1.357	−0.036	−0.709	0.479	0.775	1.290
Monthly personal income	2.727	1.428	0.092	1.910	0.057	0.863	1.159
Training in cyber ethics (yes vs. no)	−2.714	2.269	−0.055	−1.196	0.232	0.939	1.065
Average daily internet use	−0.109	1.538	−0.004	−0.071	0.944	0.790	1.265
Hospital ethical climate total score	0.542	0.099	0.273	5.496	< 0.001	0.815	1.227
Nurses’ compassion competence total score	0.232	0.090	0.129	2.579	0.010	0.803	1.246

*R* = 0.454, *R*^2^ = 0.206, adjusted *R*^2^ = 0.182, *p* < 0.001. Reference categories: education = associate degree or below; professional title = nurse; cyber ethics training = no. Years of service, weekly working hours, monthly income, and average daily internet use were entered as ordinal trend variables.

### 3.5 Structural equation model and theoretical mediation analysis

A structural equation model was constructed with hospital ethical climate as the independent latent variable, compassion competence as the mediating latent variable, and cyber ethics literacy as the dependent latent variable. Dimension scores were used as observed indicators. The model showed acceptable fit: *χ*^2^/df = 2.007, GFI = 0.964, AGFI = 0.943, TLI = 0.962, NFI = 0.946, IFI = 0.972, CFI = 0.972, and RMSEA = 0.050. The model was interpreted as a theoretical mediation model compatible with the observed cross-sectional data rather than as proof of causal mediation.

All observed indicators showed standardized loadings above 0.60. Composite reliability values were above 0.70 for all latent constructs. The AVE value for cyber ethics literacy was slightly below 0.50 but close to the conventional threshold, while CR remained acceptable; therefore, the measurement evidence was considered adequate for exploratory analysis but not sufficient to claim full independent validation of the newly developed cyber ethics literacy questionnaire (Table 5).

**Table 5:** Dimension-level measurement model reliability and convergent validity.

Latent construct	Observed indicators (standardized loadings)	CR	AVE	√AVE
Hospital ethical climate	Nurse (0.715); Patient (0.780); Physician (0.698); Administrator (0.671); Hospital (0.751)	0.846	0.524	0.724
Nurses’ compassion competence	Communication competence (0.677); Sensitivity (0.721); Insight competence (0.739)	0.756	0.508	0.713
Nurses’ cyber ethics literacy	Knowledge of cyber ethics and law (0.643); Beliefs in cyber ethics (0.697); Cyber ethical behavior (0.732)	0.733	0.478	0.692

CR, composite reliability; AVE, average variance extracted. Values were calculated from standardized dimension-level factor loadings in the structural equation model.

**Figure 2:**
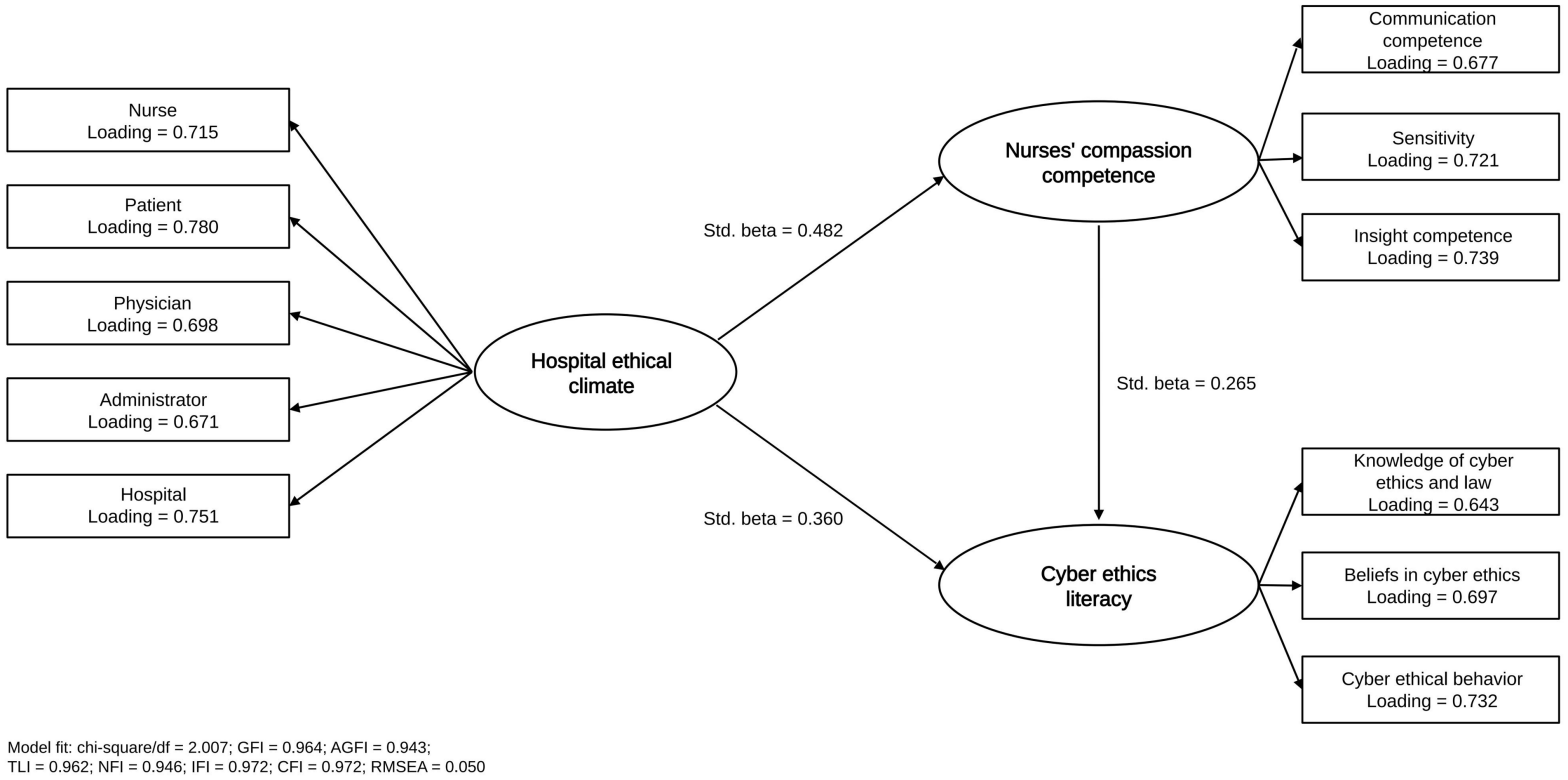
The meditation model with standardized coefficients.

The standardized path coefficient from hospital ethical climate to compassion competence was 0.482, the coefficient from compassion competence to cyber ethics literacy was 0.265, and the direct coefficient from hospital ethical climate to cyber ethics literacy was 0.360. The standardized indirect effect was 0.127 (95% CI: 0.052–0.228, *p* = 0.001), accounting for 26.08% of the total effect. Because the confidence interval did not include zero, compassion competence statistically mediated the association between hospital ethical climate and cyber ethics literacy in the proposed theoretical model (Figure 2 and Table 6).

**Table 6:** Mediation analysis of nurses’ compassion competence between hospital ethical climate and cyber ethics literacy.

Type of effect	Standardized effect	SE	95% CI lower	95% CI upper	*p*	Proportion of effect (%)
Indirect effect	0.127	0.044	0.052	0.228	0.001	26.08
Direct effect	0.360	0.078	0.202	0.510	<0.001	73.92
Total effect	0.487	0.060	0.363	0.606	<0.001	100.00

Confidence intervals were estimated using 5,000 bootstrap samples.

## 4 Discussion

This multicenter cross-sectional study examined the associations among perceived hospital ethical climate, compassion competence, and cyber ethics literacy among clinical nurses in three Grade A tertiary hospitals in Shandong Province, China. The results showed that nurses’ cyber ethics literacy was at a moderately high level and was positively associated with both perceived hospital ethical climate and compassion competence. The structural equation model further suggested that compassion competence partially mediated the association between hospital ethical climate and cyber ethics literacy. These findings are consistent with the social cognitive view that professional behavior and ethical cognition are shaped by both environmental and personal factors, but they should not be interpreted as demonstrating causality.

The overall cyber ethics literacy score was relatively high, but the lowest dimension score was knowledge of cyber ethics and law. This pattern suggests that nurses may report relatively appropriate online behaviors while still having gaps in formal knowledge about digital ethics, legal responsibility, and online professional standards. Behaviors may be more visible and more strongly shaped by organizational norms or managerial supervision than abstract legal-ethical knowledge (32). Previous research has also reported that nurses and midwives may have insufficient awareness of social media policies and online professionalism expectations (33). In the Chinese healthcare context, rapid digitalization of hospital services and growing use of online patient communication may outpace the development of structured cyber ethics education, which may explain why knowledge-related scores were lower than behavior-related scores.

Hospital ethical climate was positively associated with nurses’ cyber ethics literacy. This finding supports the view that organizational climates provide cues about acceptable professional conduct and ethical decision-making (19, 20). A favorable ethical climate may help nurses recognize that confidentiality, respect, accountability, and boundary maintenance remain important even when communication occurs through social media, telehealth, or other online platforms. However, the explanatory power of the regression model was modest (*R*^2^ = 0.206, adjusted *R*^2^ = 0.182), indicating that cyber ethics literacy is influenced by additional factors beyond those measured in this study. The result differs from some studies in which ethical climate was not significantly associated with ethical behavior, possibly because organizational expectations, hierarchical management, and digital governance practices vary across cultural and institutional contexts (34).

Compassion competence was also positively associated with cyber ethics literacy. This relationship is theoretically plausible because compassion competence involves sensitivity, insight, and communication competence, which may help nurses anticipate how online words, images, or disclosures could affect patients, colleagues, and public trust. Previous studies have linked compassion competence with patient safety competence, burnout, ethical sensitivity, and ethical behavior (35–40). Extending this literature, the present study suggests that compassion competence may also be relevant to digital nursing professionalism. Nurses with higher compassion competence may be more likely to consider patients’ vulnerability and emotional needs when navigating online interactions, thereby supporting ethical behavior in digital environments.

The mediation model indicated that compassion competence statistically mediated part of the association between hospital ethical climate and cyber ethics literacy. This finding is compatible with the social cognitive pathway of environment-personal factor-behavior. A supportive ethical climate may help nurses feel morally supported, reduce moral distress, and preserve psychological resources for compassionate practice (41–46). Compassion competence, in turn, may make nurses more attentive to patient privacy, dignity, and professional boundaries in digital communication. Nevertheless, this indirect effect was derived from cross-sectional data; therefore, the term mediation is used in a statistical and theoretical sense. Longitudinal or intervention studies are needed to confirm whether changes in ethical climate precede changes in compassion competence and cyber ethics literacy.

Several practical implications require careful interpretation. Although prior cyber ethics training was not found to be statistically significant in the regression model, this does not mean such training is unimportant. The training variable was only measured using a binary yes/no item, which failed to capture the quality, frequency, duration, content, or recency of training. Therefore, future research can adopt more comprehensive assessment methods. Furthermore, hospitals should consider integrating cyber ethics education into broader organizational ethics development, with concrete guidance on patient confidentiality, social media boundaries, online communication, misinformation, and professional accountability. Cyber ethics has also been identified as an emerging concern in health-professions education (22). International professional guidance emphasizes protecting patient privacy, maintaining professional boundaries, and following institutional social media policies (47). Therefore, future training should be standardized, scenario-based, and evaluated using more precise measures.

This study contributes to nursing ethics research by extending the discussion from traditional clinical ethics to digital healthcare ethics. It also clarifies that compassion competence is not interchangeable with empathy: compassion competence includes empathic understanding but additionally emphasizes sensitivity, insight, communication, and action-oriented professional response. By positioning compassion competence between perceived ethical climate and cyber ethics literacy, the study offers a theoretically grounded but non-causal framework for understanding how organizational ethics and individual professional capacities may jointly relate to digital nursing professionalism.

However, This study has several limitations. First, the cross-sectional design prevents causal inference and does not establish temporal order among hospital ethical climate, compassion competence, and cyber ethics literacy. The mediation analysis should therefore be interpreted as theory-compatible statistical mediation rather than causal mediation. Second, all main variables were measured using self-reported questionnaires collected at the same time, which creates risks of common method variance, recall bias, and social desirability bias. Although anonymity and voluntary participation were used to reduce response pressure, these procedures cannot eliminate common method bias. Third, convenience sampling was conducted from three Grade A tertiary hospitals in Shandong Province, China. This approach limits representativeness and reduces generalizability to nurses in other regions, hospital levels, or healthcare systems. The anonymized dataset did not retain hospital identifiers, so hospital-specific participant numbers, non-response analyses by hospital, cluster-robust standard errors, and multilevel modeling could not be performed. In this study, the hospital ethical climate score reflects the individual-level perception score of the nurses, rather than a consolidated indicator at the departmental or hospital level. Therefore, the results are limited to the individual cognitive level and cannot be generalized to organizational-level effects. Fourth, the Nurses’ Cyber Ethics Literacy Questionnaire is a newly developed instrument. Although prior reliability, content validity, exploratory factor analysis, and present dimension-level measurement evidence were reported, independent external validation and item-level confirmatory factor analysis in diverse samples are still needed. Future research should use longitudinal or intervention designs, retain hospital-level identifiers for multilevel analysis, and include objective or multi-source measures of digital professionalism when feasible.

## 5 Conclusion

Nurses’ cyber ethics literacy in this multicenter sample was at a moderately high level, with lower scores in knowledge of cyber ethics and law than in cyber ethical behavior. Perceived hospital ethical climate and compassion competence were positively associated with cyber ethics literacy, and compassion competence statistically mediated the association between ethical climate and cyber ethics literacy in a theory-driven model. These findings suggest that digital nursing professionalism may be related to both organizational ethical environments and individual compassion competence. However, because the study was cross-sectional and based on self-reported data, the findings should be interpreted as associative rather than causal. Hospitals should strengthen ethical climate construction, provide structured cyber ethics education, and support compassion competence as part of comprehensive digital ethics governance.

## Data Availability

The raw data supporting the conclusions of this article will be made available by the authors, without undue reservation.

## Footnotes

1
https://www.wjx.cn/
